# Early Phase Management of the SARS-CoV-2 Pandemic in the Geographic Area of the Veneto Region, in One of the World’s Oldest Populations

**DOI:** 10.3390/ijerph17239045

**Published:** 2020-12-04

**Authors:** Alessandro Camerotto, Andrea Sartorio, Anna Mazzetto, Milena Gusella, Ornella Luppi, Domenica Lucianò, Olga Sofritti, Cristiano Pelati, Emilia Munno, Andrea Tessari, Simone Bedendo, Margherita Bellè, Federica Fenzi, Andrea Formaglio, Annalisa Boschini, Alberto Busson, Elisabetta Spigolon, Paolo De Pieri, Paola Casson, Edgardo Contato, Antonio Compostella

**Affiliations:** 1 UOC Laboratory Medicine AULSS 5, 45100 Rovigo, Italy; milena.gusella@aulss5.veneto.it (M.G.); emilia.munno@aulss5.veneto.it (E.M.); 2 UOC Cure Primarie AULSS 5, 45100 Rovigo, Italy; andrea.sartorio@aulss5.veneto.it (A.S.); ornella.luppi@aulss5.veneto.it (O.L.); 3 Dipartimento di Scienze della Vita e Biotecnologie, Università degli Studi di Ferrara, 44121 Ferrara, Italy; anna.mazzetto@student.unife.it; 4 Direzione Funzione Territoriale AULSS 5, 45100 Rovigo, Italy; domenica.luciano@aulss5.veneto.it; 5 UOC Medicina Trasfusionale AULSS 5, 45100 Rovigo, Italy; olga.sofritti@aulss5.veneto.it; 6 UOS Direzione Professioni Sanitarie Ospedale AULSS 5, 45100 Rovigo, Italy; cristiano.pelati@aulss5.veneto.it (C.P.); simone.bedendo@aulss5.veneto.it (S.B.); 7 UOSD Microbiologia AULSS 5, 45100 Rovigo, Italy; andrea.tessari@aulss5.veneto.it; 8 UOC Servizio Igiene e Sanità Pubblica AULSS 5, 45100 Rovigo, Italy; margherita.belle@aulss5.veneto.it (M.B.); federica.fenzi@aulss5.veneto.it (F.F.); andrea.formaglio@aulss5.veneto.it (A.F.); 9 Ufficio Relazione Pubbliche e Comunicazione AULSS 5, 45100 Rovigo, Italy; annalisa.boschini@aulss5.veneto.it (A.B.); alberto.busson@aulss5.veneto.it (A.B.); 10 Polo Formativo Aziendale AULSS 5, 45100 Rovigo, Italy; elisabetta.spigolon@aulss5.veneto.it; 11 Direzione Funzione Ospedaliera AULSS 5, 45100 Rovigo, Italy; paolo.depieri@aulss5.veneto.it; 12 Direzione Generale AULSS 5, 45100 Rovigo, Italy; paola.casson@aulss5.veneto.it (P.C.); edgardo.contato@aulss5.veneto.it (E.C.); antonio.compostella@aulss5.veneto.it (A.C.)

**Keywords:** SARS-CoV-2, COVID-19, public health, pandemic management

## Abstract

The first cases of Coronavirus disease-2019 (COVID-19) were reported on 21 February in the small town of Vo’ near Padua in the Veneto region of Italy. This event led to 19,286 infected people in the region by 30 June 2020 (39.30 cases/10,000 inhabitants). Meanwhile, Rovigo Local Health Unit n. 5 (ULSS 5), bordering areas with high epidemic rates and having one of the world’s oldest populations, registered the lowest infection rates in the region (19.03 cases/10,000 inhabitants). The aim of this study was to describe timing and event management by ULSS 5 in preventing the propagation of infection within the timeframe spanning from 21 February to 30 June. Our analysis considered age, genetic clusters, sex, orography, the population density, pollution, and economic activities linked to the pandemic, according to the literature. The ULSS 5 Health Director General’s quick decision-making in the realm of public health, territorial assistance, and retirement homes were key to taking the right actions at the right time. Indeed, the number of isolated cases in the Veneto region was the highest among all the Italian regions at the beginning of the epidemic. Moreover, the implementation of molecular diagnostic tools, which were initially absent, enabled health care experts to make quick diagnoses. Quick decision-making, timely actions, and encouraging results were achieved thanks to a solid chain of command, despite a somewhat unclear legislative environment. In conclusion, we believe that the containment of the epidemic depends on the time factor, coupled with a strong sense of awareness and discretion in the Health Director General’s decision-making. Moreover, real-time communication with operating units and institutions goes hand in hand with the common goal of protecting public health.

## 1. Introduction

In January 2020, the first cases of infection of Severe Acute Respiratory Syndrome Coronavirus 2 (SARS-CoV-2) were discovered in Italy. SARS-CoV-2 is a new coronavirus, which we will discuss in Section 3.1. These cases turned out to be directly related to the Coronavirus disease-2019 (COVID-19) pandemic that was first reported in December 2019 in Wuhan, China. The first cases in the Veneto region were registered in the village of Vo’ near Padua, 40 km away from Rovigo, on 21 February. The number of cases had increased to 19,286 on 30 June, corresponding to 39.30 per 10,000 inhabitants.

For the purposes of this study, 21 February 2020 was considered to be the exact starting time (time 0), and the study aimed to understand timing with respect to epidemic spread in Veneto and parts of Italy. Data collection and analysis were conducted up until 30 June 2020, when the curve of new cases began to flatten.

The district of Rovigo consists of flat lowlands situated in the south of the Veneto region, between the Adige and Po rivers, directly neighboring districts such as Padua and Verona, where high rates of SARS-CoV-2 infection were reported. It has a population of 237,000 inhabitants, representing one of the oldest in the world, and presented significantly lower infection rates than other areas, with a total of 444 cases, corresponding to 19.03 out of 10,000 inhabitants.

The aim of this work is to describe the management of the COVID-19 pandemic in the district of Rovigo during the first phase until 30 June, and to discuss the reasons for the low prevalence observed. Furthermore, the study sets out to illustrate the “disease process”, which consists of the phases of prevention, communication, infection, diagnosis, and isolation. Therapy and clinical management are not objects of this analysis.

## 2. Materials and Methods

### 2.1. Study Site

In this manuscript, we first briefly describe the virus and its features and then deal with factors that could be associated with the low prevalence observed in the district of Rovigo compared to other Italian districts. Finally, we present pandemic management in Rovigo district.

### 2.2. Study Group

In order to evaluate and understand the effects of various factors on the spread of SARS-CoV-2 in the district of Rovigo, we decided to evaluate other districts of the Veneto region (Padua, Verona, and Venice), the Emilia-Romagna region (Ferrara, Ravenna, Modena, Parma, and Forlì-Cesena), and Lombardy (Mantua). We included the districts of Venice, Padua, Verona, Ferrara, and Mantua due to their geographical proximity to Rovigo, and likewise, the addition of Forlì, Cesena, and Ravenna, which are located on the shores of the Adriatic Sea, and Parma and Modena in the Po Valley, was justified for inclusion in the study.

### 2.3. Parameters

For each district, we selected different factors, including demographics, the number of positive cases, and the number of people placed in quarantine.

The demographics were obtained from the Italian National Institute of Statistics (ISTAT) through its open database, accessed on 14 September.

Data on positive cases were obtained from the GitHub page of The Civil Protection Department (URL: https://github.com/pcm-dpc/COVID-19).

Data relating to the number of people placed under quarantine were provided by the UOC Hygiene and Public Health Service of Rovigo Local Health Unit n.5 and obtained by the daily report of Azienda Zero. Due to initial difficulties in finding data, our team conducted analyses solely for the period between 18 March and 21 April for the districts within the Veneto region, and then added to the above-mentioned districts of Rovigo, Padua, Verona, and Venice the further three districts of Vicenza, Treviso, and Belluno. The number of people in quarantine for each case was calculated by dividing the total number of people in quarantine each day by the number of positive cases that day.

### 2.4. Statistical Analysis

We calculated Pearson’s correlation between cases per 10,000 people, the population density (defined as inhabitants per km^2^), and the business density (defined as business per km^2^). In the case of a significant correlation, we used a linear regression model to find correlations between variables, considering cases per 10,000 people as the dependent variable. All data used to calculate the Pearson’s correlation and linear regression were previously subjected to a Shapiro–Wilk test and Dixon outlier test. In order to find differences between districts in terms of people being placed under quarantine, a one-way ANOVA test was performed.

We considered a *p*-value ≤ 0.05 to be significant.

Statistical analysis was conducted with Jamovi (Version 1.2) [Computer Software], the Jamovi project (2020), retrieved from https://www.jamovi.org and Libreoffice Calc Foundation, T. D. (2020), LibreOffice Calc., retrieved from https://www.libreoffice.org/discover/calc/.

Graphs were created with Libreoffice Calc and Microsoft Excel 365 Microsoft Corporation. (2018), retrieved from https://office.microsoft.com/excel.

## 3. Results and Discussion

### 3.1. The Virus

Coronaviruses (CoV) are RNA-viruses responsible for both common cold syndromes and severe respiratory syndromes, such as SARS and Middle East Respiratory Syndrome (MERS). The spike or S glycoprotein is a transmembrane protein which enables the infection of human cells that express the angiotensin-converting enzyme 2 (ACE2) receptor.

COVID-19 is a zoonotic disease with respiratory and systemic symptoms that have spread across the globe, reaching pandemic proportions [1,2]. According to the World Health Organization (WHO), the disease spread to all five continents, with 10,185,374 cases reported up to 30 June. COVID-19 seems to be less severe than the two previous epidemics of SARS and MERS, presenting lower mortality rates. However, it is characterized by a higher transmissibility rate because of a long incubation time (up to 14 days), which facilitated its swift spread inside and outside China.

### 3.2. Infection, Disease, and Gender Differences

The most common means of human-to-human transmission for the COVID-19 virus are

- By air, through saliva and aerosol secretions from the upper airways through coughing and/or sneezing;
- By close direct contact as a result of a handshake before touching the mucous membranes of the mouth, nose, and eyes with contaminated hands;
- By the fecal–oral route.

In the 12th Situation Report of 1 February 2020, the WHO stated that COVID-19 is primarily transmitted from symptomatic patients, and although rarer, from patients who are asymptomatic with infection. The most common symptoms of an upper respiratory tract infection include a fever, cough, headache, pharyngodynia, breathing difficulties, and fatigue. Lower respiratory tract involvement and complications are more frequent in people with pre-existing cardio-vascular and/or respiratory diseases, infants, the elderly, and those with immunosuppressive conditions.

According to data from The Higher Institute of Health (ISS), a COVID-19 patient’s mean age was 63 in the time period under consideration. According to the report [1], the disease onset, clinical manifestations, responses to treatments, and outcome were significantly different among men and women due to

- The immune response, both innate and adaptive, which is more effective in women than in men [3],
- Genetics: The COVID-19 virus penetrates into cells by binding to the ACE2 receptor, which is an enzyme that regulates arterial vasoconstriction. It is expressed on the cells of the lung epithelium, where it protects the lung from damage caused by infections, inflammation, and stress. When the virus enters the cell, it reduces the ACE2 expression and therefore its protective function [4]. Female cells have two X chromosomes and to prevent the redundant expression of the products of the genes present in double copies on the X chromosomes, physiological epigenetic random inactivation of one of the two chromosomes occurs. However, some chromosomal loci escape the inactivation and can be overexpressed. ACE2 is encoded in a non-inactivated region of the X chromosome, supporting the hypothesis of a greater expression in the lungs of women, thus ensuring its protective function, even during infection [5]. Finally, it should be emphasized that the role of the TMPRSS2 serine protease, which is structurally and functionally integrated in the ACE2 receptor, increases the entry of the virus into cells almost 100-fold [6]. The expression of TMPRSS2 is androgen-dependent, thereby explaining its disadvantage in males [7].

### 3.3. ULSS 5 Population

The ageing index of the ULSS 5 population (242.4) is the highest among all the provinces of the Veneto region and above the Italian average (179.4). Compared to the rest of the world, Italy is second only to Japan with regards to its ageing index.

Rovigo district’s mean age is 48.50 years and 26.8% of the population is older than 65 years, while the Italian mean age is 45.7 years and 23.2% of the population is older than 65 years. 

The gender ratio is 0.95, which is the same as the national ratio and the other considered districts.

To better understand the spread of SARS-CoV-2 and the impact of the containment measures carried out by ULSS 5, we decided to consider nine more districts in our analysis, including Venice, Padua, Verona, Ferrara, Forlì-Cesena, Ravenna, Mantua, Parma, and Modena.

The decision to include the districts of Venice, Padua, Verona, Ferrara, and Mantua was due to their geographical proximity to Rovigo, and likewise, the addition of Forlì, Cesena, and Ravenna, which are located on the shores of the Adriatic Sea, and Parma and Modena in the Po Valley, was justified for inclusion in the study.

**Figure 1:**
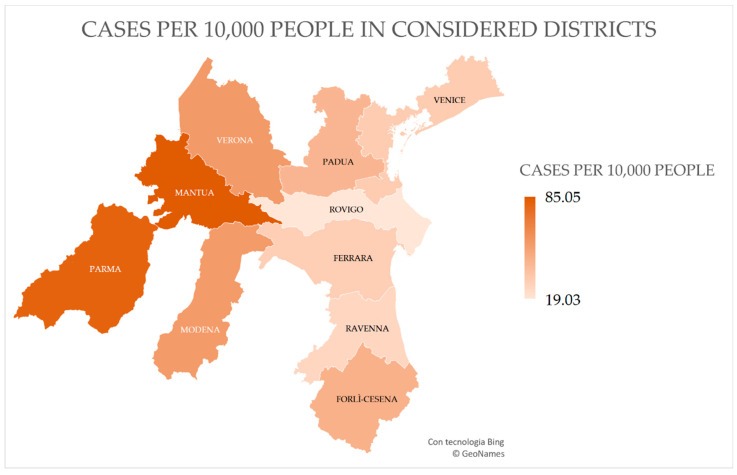
Map of districts considered in our analysis, colored according to cases per 10,000 people.

All of the data are shown in Table 1, and their representation on a map of the considered districts, including the number of positive cases per 10,000 people, is shown in Figure 1.

**Figure 2:**
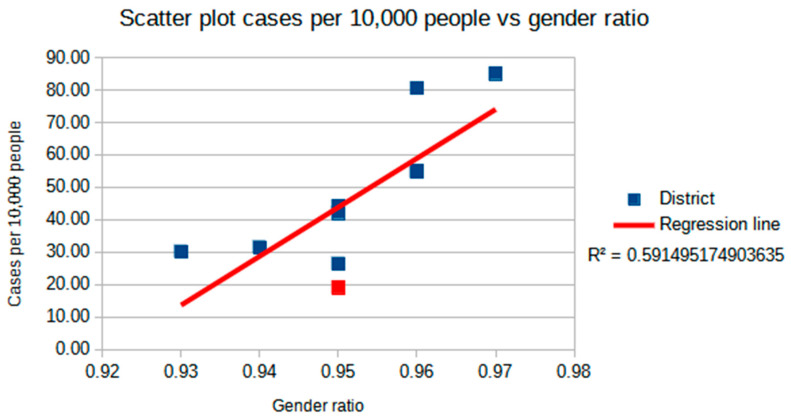
Scatter plot of cases per 10,000 people vs. the gender ratio. Squares represent considered districts and the red square represents the district of Rovigo. The red line is the regression line, and its R^2^ is reported.

Pearson’s correlation coefficient (r) between the gender ratio and the number of cases per 10,000 population was shown to be significant, with r(8) = 0.769, *p* = 0.009. This evidence was coherent with ongoing studies, which are presented in Section 3.2. A simple regression was calculated to predict the cases per 10,000 people based on the gender ratio. A significant regression equation was found (F(1.8) = 11.6, *p* = 0.009), with an R^2^ of 0.591. Although the gender ratio in the Rovigo health district is higher than other districts under consideration, the number of observed cases was lower than expected according to linear regression (Figure 2).

### 3.4. Population Genetics: Beta Thalassemia and Blood Types

According to neonatal screening and the data of blood donors from ULSS 5, collected by the Operational Unit of Blood Transfusion, trait carriers of beta thalassemia in the district of Rovigo are estimated to represent around 7.5–8% of the whole population. The time periods were 1979–2020 for neonatal and 1973–2013 for school-aged screening.

The frequency of blood types in the district of Rovigo was as follows: A+, 37.8%; A−, 4.7%; O+, 42.6%; O−, 6%; AB+, 4.3%; AB−, 0.6%; B+, 2.4%; and B−, 1.6%.

During the SARS-CoV-2 pandemic, areas with a high frequency of thalassemic patients, such as Rovigo, had a relatively low number of cases. Several studies point us toward an association between thalassemia and a lower COVID-19 incidence; however, currently, there is no definitive scientific evidence for this [8]. Despite this, some reports suggest that viral proteins could attack the heme molecule on the 1-beta chain of hemoglobin, leading to a dissociation on iron and porphyrins, seizing the latter. As a result, this mechanism inhibits the heme metabolism, which could induce a lower level of oxygen transport and anemia.

Some other analyses have demonstrated an association between SARS-CoV-2 susceptibility and blood types. Infection rates seem higher in patients with a group A blood type and lower in patients with a group O blood type [9]. The mechanism of infection of SARS-CoV-2 in blood cells is still not clear.

### 3.5. The Territory and Orography of the Province of Rovigo/ULSS 5, Pollution, and Economic Activities

The district of Rovigo is located in the Po valley, which is an area with high levels of pollution and vibrant human activity. High levels of pollution, especially PM_10_ and PM_2.5_, were previously found to be related to a high incidence of COVID-19 cases [10,11]. Furthermore, the climate and wind speed are supposedly related to increases in COVID-19 cases, due to several pieces of evidence [12,13,14].

Considering the pollution levels, wind speed, population density, and respiratory disorder rate for people, Coccia et al. proposed an index for quantifying the environmental risk of COVID-19 for 55 Italian provincial capitals [15]. Rovigo, which is the provincial capital of the homonymous district, was considered as being at high risk (19th in descending order of risk). However, this ranking was modified on 7 April to place Rovigo in 53rd place in descending order of number of cases [15]. Among the other districts, six of them were deemed to be at medium risk (Forlì-Cesena, Mantua, Parma, Ravenna, Venice, and Verona) and the remaining at high risk (Ferrara, Modena, and Padua). Nonetheless, considering cases of COVID-19 in the districts on 30 June, they all had more cases per 10,000 people than the district of Rovigo. In the district of Rovigo, from 21 February to 30 June, 46,443 swabs were carried out. In the whole Veneto region, for the same period, 975,466 swabs were carried out, so in the district of Rovigo, 4.85% of all swabs were carried out in Veneto. These data are coherent because 4.75% of the Veneto population lives in the district of Rovigo. In the considered districts located outside of the Veneto region, these data are analogues for both districts and the whole region. Guidelines for testing were common across the whole of Italy. All swabs were analyzed with a PCR-based method.

**Figure 3:**
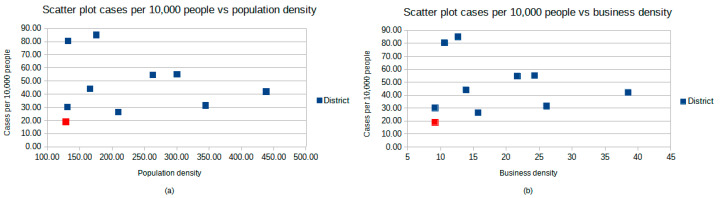
(**a**) Scatter plot cases per 10,000 people vs. the population density. Population density is defined as inhabitants per km^2^. Squares represent considered districts, and the red square represents the district of Rovigo. (**b**) Scatter plot cases per 10,000 people per business density. Business density in defined as business per km^2^. Squares represent considered districts and the red square represents the district of Rovigo.

To better understand which factors could explain the differences between the district of Rovigo and the other districts, the presence of a correlation between cases per 10,000 people and the population density (defined as inhabitants per km^2^) and business density (defined as business per km^2^) was analyzed. Using Pearson’s correlation coefficient, no correlation between cases per 10,000 people and the density of the population (r(8) = −0.105, *p* = 0.772) was found, and the same was true between the population density and business density (r(8) = −0.076, *p* = 0.836). Scatter plots of these data are shown in Figure 3.

The absence of a correlation between cases and the population density is surprising. In many studies conducted since the beginning of the SARS-CoV-2 outbreak, the population density has been considered to be related to the number of cases, mainly because a high population density can facilitate the spread of the virus [14,15,16,17,18]. However, there are also other studies that did not find a correlation between the density and cases [12,19,20,21,22]. It is presumed that a high population density can facilitate the spread of the virus due to more people engaging in social interactions to a larger extent, thereby making social distancing harder to maintain and causing public transportation to be more overcrowded. However, in more densely populated cities or counties/districts, the population is more likely to adopt social distancing measures and respect laws and regulations decided by the government than people in low density areas [21,22,23]. Finally, the pattern and characteristics of social interactions in a population are very complex and difficult to analyze and may not be directly and linearly related to the population density [22,24,25,26]. This evidence can explain why the population density in our study is not correlated with cases per 10,000 people and why districts with a lower density exhibited more cases per 10,000 people than more densely populated ones.

As a conclusion, we can assume that the low number of COVID-19 cases per 10,000 people observed in the district of Rovigo is not only due to its low population density.

Another factor that may partly explain the low incidence in the Rovigo province is the high ageing index, because younger people normally display more social interactions. However, as has been established [24], the pattern and characteristics of social interactions are very complex. For example, even if elderly people are less interested in parties or other events with many social interactions (such as concerts), they can attend other occasions involving social contact and consequently get infected; in fact, they often go to cafes or play games like cards or chess or go to their general practitioner or other hospitals more frequently compared to younger people. Furthermore, considering the strong family value for Italian people, they are often in close contact with sons and nephews, which may represent another source of contagion.

### 3.6. Timing of Events in an Unstable and Unclear Landscape of Laws and Regulations

- At 30 days before our 0 time (end of January/start of February): Active telephone surveillance and names provided to the police forces for about 50 students returning from the Chinese New Year celebrations. China had already informed students to remain in isolation and ULSS 5 immediately activated a voluntary isolation procedure of 14 days, despite the absence of regulatory norms by the region or the ministry;
- At 30 days (28 January): First letter to schools to activate the passive surveillance of Chinese students who had returned from risk areas (the first regional provision in this sense was on 11 February);
- +1 (22 February): Even in the absence of precise indications on isolation, all subjects of the district of Rovigo who had passed through Vo’ or Schiavonia hospital were placed in isolation. At the end of February, 220 subjects had been placed in solitary confinement;
- +2 (23 February): A systematic epidemiological investigation began with the isolation of all close and not close contacts of positive subjects (first contact). On 30 June, there were 19,286 SARS-CoV-2 positive cases in the Veneto region, of which 444 were from the district of Rovigo, with 51 subjects in home isolation. The number of swabs performed from 21 February to 30 June was 46,443.

For each person testing positive following a swab, all close contacts were put into quarantine. In the district of Rovigo, in the period spanning from 2 February to 6 June, 3212 persons were self-quarantined in their homes, with a mean of 7.17 people in quarantine for each positive case (also considering the positive patients). Comparing this value with other districts is not easy due to the lack of public data referring to a single district. Therefore, we were only able to obtain data on the other districts of the Veneto region for the period from 18 March to 21 April, collected by Azienda Zero. In that period, for each positive patient, a mean of 4.58 people were put in quarantine in the district of Rovigo, σ^2^ = 5.26 (also considering the positive patients). On the contrary, in the other districts (Padua, Verona, Venice, Vicenza, Treviso, and Belluno), the mean was 2.27 people per positive patient, σ^2^ = 0.44. Using a one-way ANOVA statistical analysis, a significant difference (*p* ≤ 0.00001) was found between the number of people placed under quarantine in the district of Rovigo and the other districts of the Veneto region.

### 3.7. Integrated Approach of Activities: Public Health, Territorial Assistance, and Nursing Homes

At +6 days (26 February), there was a meeting with nursing home (RSA) risk self-assessment managers, nursing homes were closed to outsiders, and monitoring and screening procedures commenced.

At +18 days (10 March), there was a reorganization of hospital admissions, with compulsory booking and social distancing strategies put into place.

Curbing of the infection in nursing homes was the result of a combination of different strategies, including governance, timeliness, and integration.

#### 3.7.1. Governance

Planning of meetings with all RSA managers: Nine meetings from 2 February to 7 July 2020.

On 7 April (+46 days), a working group was created, composed of three RSA Directors, the Health Director General, and the Health & Social Care Services Director of ULSS 5: Five meetings up to 10 July 2020.

#### 3.7.2. Timeliness

The timeliness of the highest impact measures was decisive: The closure of structures to visitors and family members was decided on 22 February (+1 day from Vo’, −24 days from regional indications) and ended on 9 March 2020.

The activation of a multidisciplinary risk-assessment team on 18 March, all RSAs (+26 days from Vo’, −12 days from regional indications), and the first processing of a Public Health Plan (PSP) took place on 1 April (+40 days from Vo’, +1 day from regional indications).

Early screening was achieved by performing 15,603 diagnostic tests (various types) on 2484 guests in the period 2 February–10 July 2020, corresponding to 6.3 per person in 140 days and around 1 test every 22 days for each person, from the beginning of the pandemic. It is important to note that the availability of tests was too limited and screening on a large scale had not yet been foreseen in that period.

#### 3.7.3. Integration

The multidisciplinary team (composed of doctors, nurses, and prevention technicians) was supported by other professional profiles in line with regional indications, working closely with the Disability and Non-Self-Sufficiency Unit, Nursing Professions Management, and the Training Center. These professionals provided knowhow and expertise in the realm of organizational and assistance dynamics within this specific context. Their input considerably improved the appropriateness and effectiveness of the interventions.

The following actions were also performed:

- The “immediate availability” of the professionals mentioned above, in synergy with healthcare professionals already in place in the Hygiene and Public Health Service;
- The integration of a nursing coordinator from the ULSS within the settings affected by epidemic outbreaks, in order to offer local support in emergency management;
- Specific training, e-learning (distance learning and online training) or face-to-face training sessions, organized where required, provided by the ULSS 5 Training Center;
- Additional and customized supplies of personal protective equipment (PPE), according to needs.

In the district of Rovigo, there are 23 nursing homes for elderly people with a total of 2097 residents, 1 retirement home for elderly people with 91 residents, and 6 nursing homes for disabled people with a total of 341 residents, resulting in a total of 2529 residents and 1897 health and social care operators.

In the period between 20 February and 10 July 2020, a total of 112 residents among seven elderly nursing homes tested positive for SARS-CoV-2, of which 11 died from COVID-19. Among the operators, on the other hand, 37 people tested positive, with no cases of death.

Upon closer inspection, four of the 23 nursing homes (17.39%) for elderly people experienced cases of COVID-19, with a total of nine infected residents out of 2097 total residents (0.43%) and a mean of 2.25 infected residents per structure. Five of the residents (0.24%) died of COVID-19, with a mean of 1.25 deaths per structure and a case fatality rate (CFR) of 55.55%.

Considering the data presented in other studies [27,28,29,30,31,32,33,34,35] conducted in nursing homes for elderly people, we would have expected a significantly higher number of cases, and consequently, a higher number of deaths.

The combined effect of isolation measures, widespread testing of all residents, and the implementation of infection prevention and control (IPC) activities that were all promptly put in place in the district resulted in an effective reduction in the spread of the virus inside its nursing homes [29,30,36].

Conversely, the CFR was higher than that encountered in other reports [27,28,37], even if this datum was affected by the very limited number of cases observed. The death ratio is lower than that of the survey conducted by the Italian Higher Institute of Health (I.S.S.) [38].

Among the nursing homes for disabled people in the study, 96 out of 341 (28.15%) residents tested positive in two structures out of six (33%), with a mean of 48 cases per structure. Two of the residents (so 0.59%) died of COVID-19, with a mean of 1.25 deaths per structure and a case fatality rate (CFR) of 2.09%. Because the residents of these structures represent a more uneven population, due to the different types of disability and the medical conditions that it presents, it was difficult to make a comparison with other studies.

### 3.8. ULSS 5’s Integrated Approach with “External” Institutions

At +1 day (22 February), there was a meeting of institutional bodies (Prefecture, Police Headquarters, Carabinieri Command, President of the Social Health Conference, President of the District Committee, and Public Health Service) in the ULSS 5 head office, in order to agree to unitary and shared coordination for managing the pandemic.

At +3 days (24 February), general practitioners (GPs) and the pediatricians of free choice (PFCs) held a committee meeting to agree to unitary and shared coordination for managing the pandemic.

At +4 days (25 February), a conference of mayors and a meeting of pharmacists was organized to agree to unitary and shared coordination for managing the pandemic.

### 3.9. External Communication towards Population Management and Internal Organization

ULSS 5 Strategic Management invested in digital communication, adding an Instagram page to the two existing social network channels of Facebook and YouTube, in order to reach a younger target population. All three channels were labeled in the same way (“SaluteUlss5”), in order to give a uniform appearance to ULSS 5 and to facilitate their research.

From time zero (21 February 2020), Facebook’s direct function was used to livestream press conferences.

From 17 March (+27 days from time zero) and at a rate of 7 days per week, the Director General streamed a daily report on the pandemic live on Facebook and presented information about the correct rules to keep and the evolution of the pandemic to the population. Facebook guaranteed the highest number of followers with the widest age range.

The mean number of views was about 8000 people (with a minimum of 3947 and a peak of 13,623, on 25 March), considering both views during the live streams and time-delayed viewings. Facebook videos were also directly transmitted by online local newspapers, greatly amplifying the communicative wave.

After a reduction in the number of cases, from 18 May, the conference took place on a weekly basis.

In Phase 2 (4 May, +68 days from time zero), considering the reduction of the target age to whom the communication was directed (more young people were testing positive due to infections occurring in social environments such as parties and social dinners, which were more frequent in that period due to summer weather and holidays), Instagram was added to Facebook with remarkable success and high views data.

Finally, from 21 February, 4000 messages or comments with information requests arrived on ULSS 5 social channels, for which rapid feedback was provided, even outside working hours (during evenings or at weekends).

The goal of these procedures was to inform and reassure the population by means of a direct, rapid, and easy-to-access communication strategy, in order to increase compliance of the population itself in terms of politics and decisions taken.

The district of Rovigo’s population joined the national, regional, and local indications, and to date, there have been no interventions by law enforcement to disperse unauthorized crowds.

Another indirect indicator of the correct behavior of the population of the Rovigo district is represented by the ratio of people who tested positive after serological tests for SARS-CoV-2. In fact, according to the study conducted in Italy by ISTAT [39], 2.5% of the national population had immunoglobulins specific for the virus; in the Veneto region, the rate was 1.9% and in the district of Rovigo, it was 1.59%. This particularly low ratio shows that the virus circulated less than in the rest of the Veneto region or in Italy as a whole. Therefore, we can conclude that the studied population respected the advice and the dispositions of ULSS 5 and the Italian government. Finally, these data are coherent with the lower number of cases per 10,000 that we observed in the district of Rovigo and confirm that the low number of cases is not due to a low diagnostic capability.

Furthermore, the Directorate General used WhatsApp as a fast “formal” institutional communication tool, in order to accelerate the transmission of the indications that were released gradually to the public.

### 3.10. Strong “Military” Command Chain and Change in the Organizational/Productive Model

The Directorate General took full power, continuously coordinating with the other institutions and the healthcare structures of ULSS 5 and the territory, assuring a 24/7 presence in the management and transmission of directives, in order to give responses about the achievement of results and to monitor critical issues.

### 3.11. Personal Protective Equipment (PPE) Management

−15 days from time zero (6 February): Organization of a task force with general practitioners and pediatricians of free choice for the coordination of PPE management.

+1 day (22 February): First alert from Pharmaceutical Service because of information system overload due to the high demand for PPE.

At that time, it was decided to block direct orders of PPE from warehouses and to define a PPE rational quota layer, managed by the “Prevention Protection Services, and the Directorate of Professions, Medical Management and Board of Directors”.

Twice a day at 8.00 and 19.00, a PPE inventory was made and according to the demand, quantities were authorized to be distributed by 21:00 of the same quota. The double inventory time was crucial to ensuring the number and type of available PPE, in order to better plan their management in response to epidemic peaks.

The service was guaranteed 7 days a week by a multidisciplinary team that was able to send a supply of PPE on time within 48 h, on the advice of the Directorate General, depending on the needs.

Continuous support was guaranteed by monitoring and providing training and information to each healthcare facility that requested the PPE, thereby enabling 48 h of autonomy from the delivery time.

In order to empower and oversee the PPE distribution, a custody chain was activated in some sectors.

### 3.12. Molecular Diagnostics as Routine and Rapid 24 h Response

At time zero (21 February), ULSS 5 was not able to perform any kind of laboratory diagnosis for COVID-19 (molecular, antibodies, and antigenic).

In the first month of the emergency, molecular nasopharyngeal swabs were sent to the laboratory of Padua Local Health Unit and the time of response (TAT) ranged from 1 to 10 days, which was too long to satisfy the need for a rapid diagnosis and positive isolation.

The activation of diagnostics in loco aimed at obtaining an adequate TAT for the clinical and organizational needs of rapid diagnosis, therapy, and isolation.

Until 30 June, action was taken in three areas:

1. Standard molecular diagnostics with 24 h TAT;
2. Rapid molecular diagnostics with 4 h TAT;
3. Serological tests for antibody research.

#### 3.12.1. Standard Molecular Test

At +15 days, the existing laboratory of human genetics, located in Trecenta Hospital, 36 km from the Hub Hospital of Rovigo, was rapidly adapted to perform viral analyses of nasopharyngeal swabs. For viral nucleic-acid extraction, a column-based manual kit (QIAamp Viral RNA Mini Kit, QIAgen) was adopted and the CE-IVD RT-PCR commercial kit and the REALQUALITY RQ-2019-nCoV kit (AB ANALITICA, Padua, Italy) produced by a local company, that could ensure prompt delivery, were evaluated and validated on the ABI7500 Real-time PCR machine (Applied Biosystem, Foster City, CA, USA). In the space of a week, the lab obtained authorization from the Regional Reference Laboratory to analyze patients’ swabs and thereafter, from 23 March (+30 days), the lab started regularly working on COVID-19 samples. Subsequently, in order to increase the sample load capacity, other equipment was acquired: The Liaison MDX/Simplexa COVID-19 Direct system (Diasorin, Saluggia, Italy) for rapid molecular analysis; the Real-time PCR instrument CFX-96 (Bio-Rad, Hercules, CA, USA) for standard molecular analysis; and an automatic system for nucleic acid extraction (Chemagic 360, PerkinElmer, Waltham, MA, USA). From the very beginning (+31 days), personnel of UOC Laboratory Medicine, consisting of six technicians and two biologists, were assigned to the lab and trained to a high level of expertise. In the subsequent months, two other technicians and two biologists joined the group to complete the COVID-19 staff, which currently consists of 14 people and allows the lab to work from 7.00 to 21.00, 7 days a week. After one month, 250 nasopharyngeal swabs had been analyzed daily, which gradually increased to around 800 samples per day after 6 months.

#### 3.12.2. Rapid Molecular Test

At +68 days (4 May), a new lab named H24 was activated, aimed at analyzing swabs for patients who underwent the hospital’s admission process (4 h TAT); technicians from Laboratory Medicine, Microbiology, Transfusion Medicine, and Pathological Anatomy were dedicated to this work, with a consequent reorganization of services and staff.

At +82 days (18 May), new technicians were acquired on contract and subsequently on a fixed-term basis for the H24 swabs laboratory.

New instruments were purchased: Direct System Diasorin Liason MDX (for the detection of ORF1ab and S viral genes) and Cepheid Xpress (for the detection of N2 and E genes).

At +88 days (24 May), the building was set up with a new Molecular Biology laboratory facility for COVID-19 in the hub hospital of Rovigo and set about reorganizing and adapting spaces and logistics.

#### 3.12.3. Serological Diagnostics

At +42 days (4 April), rapid immunochromatographic serological tests on capillary blood were acquired and implemented; the laboratory unit managed the training of hospital and territory personnel, storage and supply of medical cards, and lancing devices in the operation units (OUs), as well as manual reporting.

From April to June, 11,020 cards were analyzed, obtaining a global prevalence of positive cases (IgG + IgM) of 1.17%.

At +120 days (23 June), serological tests for IgG and IgM quantification, Maglumi 800, Medical System, dedicated to hospital staff were carried out one time, in collaboration with the Universities of Padua and Verona.

At +114 days (17 June), in collaboration with the Italian Red Cross, a project for ISTAT estimation of the COVID-19 seroprevalence was organized by analyzing 259 tests with three positive cases, giving a global prevalence (IgG + IgM) of 1.59%, which was lower than in the other Veneto areas, as stated in Section 3.8.

## 4. Conclusions

We are aware that our scientific and managerial theories are by no means conclusive, due to the current unprecedented situation regarding a totally new pathology, which is a first in the history of *Homo sapiens*, and of which we do not fully know the pathogenetic mechanisms of infection, disease, and recovery. In fact, even if humanity, in its history, faced many pandemics, this is the first sustained by coronavirus, since SARS and MERS remained epidemic. Furthermore, the actual social and demographic features of the world are completely new and have resulted in a bigger and without precedence challenge.

Our aim was to represent an experience and provide hypotheses to explain the success in the local containment of the pandemic in our area, due to believing that the comparison of experiences is the best viaticum for facing SARS-CoV-2.

As stated in Section 3.4, although the territory of ULSS5 has a population and business density that is lower than other areas, these elements do not seem to be related to the low levels of the registered prevalence.

Similarly, statistics and literature suggest that genetic features of the population (microcythemia and blood groups) do not significantly influence the infection curve.

On the other hand, some parameters, such as the ageing index, gender ratio, blood group types, and pollution, should have been disadvantageous in terms of infectivity for the studied population.

The exclusion of these variables leads to the deduction that the “time factor” was the decisive element for the containment of the pandemic, representing an important factor, especially in the smart management of test-trace-isolating procedures.

Furthermore, the timeliness of decision-making and the high levels of discretion in providing feedback on actions, data, errors, and successes, as managed by the Directorate General Management and, in turn, by OUs in close coordination with territorial healthcare services, may have made all the difference.

In Table 2, with the time marked by time zero (21 February), this conclusion is made clear and summarized numerically.

Based on the speed required in pandemic management, we consider the following points to be vital:

- Chain of command in centralized Directorate General Management with “military-style” characteristics: Cascade transmission of decisions with rapid changes by hospital/territorial organizations in the face of data and situations that gradually followed one another;
- Ability of the Directorate General Management to inform and involve local structures/institutions in decisions quickly and consistently;
- Management’s ability to “stay on course”, align the organization on decisions, and translate the numerous regional and national regulations into concrete and clear actions;
- Use of the fastest and simplest digital tools for the transmission of directives to the OUs and territorial units to spread awareness and reach as many people as possible on the outside;
- Ability of healthcare workers to understand the singularity of the emergency by accepting directives that did not respect the classic formal bureaucratic model of the public administration in terms of the content and distribution.

Although our analysis is limited to the initial phase of the pandemic, the current data from Veneto, Italy show that the actions initially undertaken and structurally consolidated have enabled the district of Rovigo to maintain a positive situation during the pandemic [40].

In conclusion, and summing up using an image, a series of quick decisions/actions have consolidated a defense mechanism against the pandemic through the rapid diagnosis, isolation, and treatment of symptoms.

This experience has clearly demonstrated that the district public health mechanism can be effective in the management of a pandemic, limiting cases and consequently, deaths, when managed efficiently and rigorously, expressing all the capacity and potential that a Nation and Regional Health System can and must have.

## Figures and Tables

**Table 1:** Considered districts with the selected variables, with units of measurement reported in brackets.

District	Inhabitants (N.)	Men (N.)	Women (N.)	Gender Ratio	Area (km^2^)	Population Density (Inhabitants/km^2^)	Mean Age	Percentage of Population Aged 65 and over (%)	Ageing Index	Confirmed Cases at 06.30 (N.)	Cases per 10,000 People (N.)	Business per km^2^ (N.)
ROVIGO	233,366	113,665	119,721	0.95	1819.35	128.27	48.5	26.4	242.4	444	19.03	9.163163
PADUA	939,672	458,805	480,867	0.95	2144.15	438.25	45.8	22.8	175.8	3954	42.08	38.50897
FERRARA	344,840	166,031	178,809	0.93	2635.12	130.86	49.2	28.2	260.6	1040	30.16	9.165048
VERONA	930,339	456,445	473,894	0.96	3096.39	300.46	45.1	22.2	161.9	5127	55.11	24.28828
PARMA	453,930	222,062	231,868	0.96	3447.48	131.67	45.8	23.2	176.1	3657	80.56	10.62428
FORLI’ CESENA	394,833	192,398	202,435	0.95	2378.4	166.01	46.5	24.5	189.1	1740	44.07	13.88539
MANTUA	411,062	202,127	208,935	0.97	2341.44	175.56	46.2	23.9	182.3	3496	85.05	12.65802
RAVENNA	389,634	189,505	200,129	0.95	1859.44	209.54	47.4	25.5	205	1030	26.44	15.71548
VENICE	851,663	413,709	437,954	0.94	2472.91	344.4	47.1	24.8	204.5	2682	31.49	26.11256
MODENA	707,292	346,686	360,606	0.96	2688.02	263.13	45.5	23.0	168.6	3873	54.76	21.67841

**Table 2:** Summary of all actions taken by Rovigo Local Health Unit n. 5 (ULSS 5). For each action, the date and time compared to 0 time (21 February) are shown.

Timing in Chronological Order	Decisions Made by ULSS 5
28 January (−25 days)	Active surveillance of Chinese students returned from Chinese New Year celebrations.
6 February (−15 days)	Task force with family doctors and pediatricians of free choice.
21 February (0 time)	First cases of SARS-CoV-2 infection in Vo’.
22 February (+1 day)	Vo’ Euganeo becomes a red zone. All those who have passed through the outbreak areas are immediately placed in isolation. Timely closure of RSAs to outsiders. Meeting and coordination with institutional bodies. Contingent distribution of PPE.
24 February (+3 days)	Committee of general practitioners and pediatricians for unitary and shared coordination for management of the pandemic.
25 February (+4 days)	Conference with mayors and meeting with pharmacists for unitary and shared coordination for management of the pandemic.
26 February (+6 days)	Start of monitoring of older homes.
2 March (+10 days)	At Trecenta hospital, a genetics laboratory is adapted into a laboratory for the analysis of nasopharyngeal swabs using molecular diagnostics.
10 March (+18 days)	Reorganization of hospital withdrawal centers with booking obligation and social distancing.
18 March (+26 days)	Identification of COVID-19 contact for all RSAs in the area.
20 March (+27 days)	Start of Facebook streaming by the Director General to share the bulletin on the evolution of the pandemic.
23 March (+30 days)	The COVID-19 laboratory of Trecenta obtains the authorization general reference laboratory to analyze swabs; the analysis capacity is supplemented by the purchase of new instruments.
24 March (+31 days)	Reorganization of the COVID-19 staff at the Trecenta laboratory.
1 April (+40 days)	First creation of a Public Health Plan (PSP).
4 April (+42 days)	Beginning of rapid immunochromatographic serological tests on capillary blood.
7 April (+46 days)	Establishment of a small working group with three representatives of the directors of RSAs, the General Manager, and the Director of Social and Health Services.
4 May (+68 days)	24 h laboratory creation for swab analysis.
18 May (+82 days)	Hiring of new technicians for swab analysis in the laboratory h24.
24 May (+88 days)	Building works begin for a new molecular biology laboratory with a COVID-19 area.
17 June (+114 days)	ISTAT serum prevalence assessment in collaboration with the Italian Red Cross.
23 June (+120 days)	Serological diagnostics on hospital operators.
30 June (+127 days)	Contagion curve stable, with no new cases.
